# Multistatic Integrated Sensing and Communication System Based on Macro–Micro Cooperation

**DOI:** 10.3390/s24082498

**Published:** 2024-04-13

**Authors:** Xiaoyun Wang, Zixiang Han, Jing Jin, Rongyan Xi, Yajuan Wang, Lincong Han, Liang Ma, Mengting Lou, Xin Gui, Qixing Wang, Guangyi Liu

**Affiliations:** Future Research Laboratory, China Mobile Research Institute, Beijing 100053, China; wangxiaoyun@chinamobile.com (X.W.);

**Keywords:** 6G, cooperative sensing, ISAC, macrosite, microsite, multistatic

## Abstract

A novel multistatic integrated sensing and communication (ISAC) system based on macro–micro cooperation for the sixth-generation (6G) mobile network is proposed. Instead of using macrosites at both the transmitter and receiver sides, microsites are considered as receivers in cooperative sensing. This system is important since microsites can be deployed more flexibly to reduce their distances to the sensing objects, providing better coverage for sensing service. In this work, we first analyze the deployment problem of microsites, which can be deployed along the radius and azimuth angle to cover macrosite cells. The coverage area of each microsite is derived in terms of its position in the cell. Then, we describe an efficient estimating approach for obtaining the position and velocity of sensing objects in the macrosite cell. By choosing multiple microsites around the targeted sensing area, joint data processing with an efficient optimization method is also provided. Simulation results show that the multistatic ISAC system employing macro–micro cooperation can improve the position and velocity estimation accuracy of objects compared to systems employing macrosite cooperation alone, demonstrating the effectiveness and potential for implementing the proposed system in the 6G mobile network.

## 1. Introduction

Mobile networks have been revolutionized over the past few decades, evolving from providing only voice services to supporting high-speed data transmissions for massive numbers of users. The forthcoming sixth-generation (6G) mobile network is envisioned as an integration of various capabilities and technologies, including communication, sensing, computing, artificial intelligence (AI), security, big data, etc. [1,2,3]. Particularly, sensing is regarded as the key enabler in extending the capabilities of mobile networks from delivering information to perceiving the environment and providing services for novel scenarios, such as unmanned aerial vehicles (UAVs) and vehicle-to-everything (V2X), that are expected in 6G [4,5]. When combined with reconfigurable intelligent surface (RIS) or Terahertz technology [6,7,8,9], the application scenarios can be further expanded into wireless power transfer [10] and low-orbit satellites [11]. Conventional radar sensing systems share many similarities with mobile communication systems in terms of radio-frequency (RF) hardware, spectrum usage, and waveform design. Therefore, a unified dual-functional system that supports both radar sensing and mobile communication is proposed, which is referred to as an integrated sensing and communication (ISAC) system [12,13]. In addition to the benefits of resource sharing through integration, sensing and communication can achieve cooperative gains by fully exploiting the results obtained from each function [13]. For example, sensing results such as object position can aid the channel estimation or beamforming in communication [14,15], whereas the communication process can deliver the initial sensing results to various sensing nodes for speeding up the sensing process when initially activating sensing service [16,17,18]. These advantages make the ISAC system a hot topic and a desirable technology for mobile networks.

To implement the ISAC system in a mobile network, one approach is to utilize a single base station (BS) as a transceiver to detect the objects in the environment, which is referred to as a monostatic ISAC system [19,20]. In this case, the BS that transmits the sensing signals receives the reflected signals from the sensing objects at the same time. However, this simultaneous signal transmission and reception requires a full-duplex BS, which is currently an immature technology [16,21]. To avoid this issue, an additional receiver co-located with the original transmit antennas on the BS can be implemented exclusively for sensing functions. However, high isolation between the transmitter and the sensing receiver becomes necessary to suppress self-interference (SI) power due to BS self-transmission [22,23,24]. As a result, the cost, weight, size, and complexity of the BS increase considerably. Therefore, it is a great burden for operators to implement a monostatic ISAC system in mobile networks.

To avoid the issues with the monostatic ISAC system, the bistatic ISAC system, which utilizes two BSs to perform cooperative sensing, has been proposed, where one BS acts as a transmitter while the other one acts as a receiver [25,26,27]. Due to the spatial separation of the transmitter and receiver, the SI issue is overcome, and hardware modifications for a full-duplex BS are avoided. However, in the bistatic ISAC system, there is a blind zone near the line-of-sight (LoS) path between the transmitter and receiver, where the position estimation error is huge [28,29]. In addition, similar to the monostatic ISAC system, the bistatic ISAC system is unable to fully recover velocity information [28,29,30]. As a further extension of the bistatic ISAC system and to overcome these challenges, the multistatic ISAC system has been proposed, where multiple BSs form a sensing cluster for sensing service [31]. Conventional multistatic ISAC systems utilize cellular macrosites as transmitters and receivers, leveraging their large transmit power [32,33] and antenna array gain [34,35] to cover broad cells. However, employing multiple macrosites in cellular networks to perform sensing functions at the same time is not highly practical for operators. One reason is that macrosites normally have fixed positions and low density in cellular networks, making it difficult to select appropriate macrosites for cooperative sensing [32,33]. The low density also results in larger estimation errors due to the long distances between objects and macrosite receivers. Another reason is that macrosites would have different uplink and downlink working statuses in cooperative sensing, violating the principle of maintaining the same working status across the whole cellular network, thereby affecting communication services for a large number of users.

Therefore, to overcome the difficulties in multistatic ISAC systems using macrosite cooperation and to take advantage of the flexible and denser deployment of microsites, which shortens the distance to sensing objects, we propose a novel multistatic ISAC system based on cooperation between macrosites and microsites. In this system, only some microsites need to change their working status for sensing purposes, without affecting the network in a large area. For a targeted sensing area, various nearby microsite receivers, together with the central macrosite, form a sensing cluster, and their sensing results can then be jointly processed to improve sensing performance. The main contributions of this work are summarized as follows:

(1) A multistatic ISAC system with macro–micro cooperation is proposed. The proposed system makes use of flexibly deployed microsites to perform multistatic sensing with the macrosite within the cell.
(2) The deployment of microsites within the macrosite cell is investigated in terms of the channel gain for cooperative sensing.
(3) An efficient approach with joint data optimization for estimating the position and velocity of sensing objects in three-dimensional (3D) environments is described.
(4) The effectiveness of the proposed multistatic ISAC system is demonstrated by simulating the estimation errors for position and velocity. It is shown that the multistatic ISAC system using macro–micro cooperation can effectively improve object estimation accuracy compared to systems using macrosite cooperation alone. The microsite configuration with high-cost performance is also provided.

Organization: Section 2 formulates the multistatic ISAC system model. Section 3 investigates the deployment of microsites within the macrosite cell. Section 4 presents the joint optimization method used to obtain the position and velocity of objects in the proposed multistatic ISAC system. Section 5 provides simulation results to demonstrate the effectiveness of the proposed multistatic ISAC system. Section 6 discusses the challenges of the proposed system. Section 7 concludes this work.

Notations: Upper- and lower-case bold letters denote vectors and matrices, respectively. Upper-case letters in calligraphy denote sets. Letters not in bold font represent scalars. $\left|a\right|$ refers to the modulus of a scalar *a*. $\langle a,b\rangle$ refers to the inner product of two vectors, a and b. $∥a∥$ refers to the $l_{2}$-norm of vector a. $A^{T}$ and $A^{H}$ refer to the transpose and conjugate transpose of matrix A, respectively. C denotes the complex number set. $i=\sqrt{-1}$ denotes the imaginary unit.

## 2. System Model

### 2.1. Multistatic ISAC System

Consider a multistatic ISAC system with $\left(K+1\right)$ nodes, where one node (numbered zero with subscript k=0) acts as the transmitter, and the other *K* nodes (numbered 1 to *K* with subscript k=1,2,…,K) serve as the receivers. The node of the transmitter has $N_{0}$ antennas, and the node of the *k*th receiver has $N_{k}$ antennas for k=1,…,K. Utilizing the orthogonal frequency-division multiplexing (OFDM) scheme, the transmitted signal on the *n*th transmit antenna, $n=1,2,\ldots ,N_{0}$, in the baseband can be written as [36,37]

(1) $$x_{n}\left(t\right)=\sum_{n_{s}=0}^{N_{s}-1}\sum_{n_{c}=0}^{N_{c}-1}s_{n}\left(n_{c},n_{s}\right)e^{i2\pi f_{\Delta }n_{c}t}g\left(t-n_{s}T_{s}\right),\;n=1,2,\ldots ,N_{0},$$

where $N_{c}$ and $N_{s}$ denote the number of subcarriers and OFDM symbols, respectively; $s_{n}\left(n_{c},n_{s}\right)$ is the digital symbol modulated on the $n_{c}$th subcarrier of the $n_{s}$th OFDM symbol at the *n*th transmit antenna; $f_{\Delta }$ and $T_{s}$ refer to the subcarrier spacing and the OFDM symbol period including the cyclic prefix; and $g\left(t\right)$ is the pulse shaping function.

We write $s\left(n_{c},n_{s}\right)={\left[s_{1}\left(n_{c},n_{s}\right),s_{2}\left(n_{c},n_{s}\right),\ldots ,s_{N_{0}}\left(n_{c},n_{s}\right)\right]}^{T}\in C^{N_{0}\times 1}$ to collect transmitted frequency-domain symbols into a vector. After matched filtering and fast Fourier transform (FFT), the received symbols at the *k*th receiver side, k=1,2,…,K, in the frequency domain can be obtained as

(2) $$\begin{array}{cc} y_{k}\left(n_{c},n_{s}\right)= & H_{k}\left(n_{c},n_{s}\right)s\left(n_{c},n_{s}\right)+n_{k}\left(n_{c},n_{s}\right)+i_{k}\left(n_{c},n_{s}\right) \end{array}$$

where $y_{k}\left(n_{c},n_{s}\right)={\left[y_{k,1}\left(n_{c},n_{s}\right),y_{k,2}\left(n_{c},n_{s}\right),\ldots ,y_{k,N_{k}}\left(n_{c},n_{s}\right)\right]}^{T}\in C^{N_{k}\times 1}$ collects the received frequency-domain symbols at the *n*th receive antenna $y_{k,n}\left(n_{c},n_{s}\right)$ for $n=1,2,\ldots ,N_{k}$, whereas $n_{k}\in C^{N_{k}\times 1}$ and $i_{k}\in C^{N_{k}\times 1}$ are the additive noise and interference signal, respectively. The frequency-domain channel $H_{k}\left(n_{c},n_{s}\right)\in C^{N_{k}\times N_{0}}$ is given by

(3) $$\begin{array}{cc} H_{k}\left(n_{c},n_{s}\right) & =\sum_{j=1}^{J}\sqrt{\kappa_{k,j}}e^{i2\pi T_{s}f_{D,k,j}n_{s}}e^{-i2\pi \tau_{k,j}f_{\Delta }n_{c}}a_{R,k}\left(\Omega_{R,k,j}\right)a_{T}^{T}\left(\Omega_{T,j}\right) \end{array}$$

where *J* denotes the number of paths with sensing objects, $\tau_{k,j}=\frac{d_{k,j}}{c}$ is the propagation time of the *j*th path, and $d_{k,j}$ and *c* refer to the propagation distance and light speed, respectively. Specifically, $d_{k,j}=d_{T,j}+d_{R,k,j}$, where $d_{T,j}$ and $d_{R,k,j}$ are the propagation distances from the transmitter to the *j*th object and from the *j*th object to the *k*th receiver [30]. Moreover, $f_{D,k,j}=\frac{v_{\|,k,j}f_{c}}{c}$ is the Doppler frequency due to the movement of the *j*th object, where $v_{\|,k,j}$ and $f_{c}$ refer to the Doppler velocity and carrier frequency, respectively. In particular, the Doppler velocity $v_{\|,k,j}$ is given by

(4) $$v_{\|,k,j}=\frac{\partial d_{T,j}}{\partial t}+\frac{\partial d_{R,k,j}}{\partial t}=v_{\|,R,k,j}+v_{\|,T,j},$$

where $v_{\|,R,k,j}=\frac{\partial d_{R,k,j}}{\partial t}$ and $v_{\|,T,j}=\frac{\partial d_{T,j}}{\partial t}$ are the radial velocities of the *j*th object with respect to the *k*th receiver and the transmitter [30]; $a_{R,k}\left(\Omega_{R,k,j}\right)\in C^{N_{k}\times 1}$ and $a_{T}\left(\Omega_{T,j}\right)\in C^{N_{0}\times 1}$ are the steering vectors of the receive antennas and the transmit antennas, respectively; and $\Omega_{R,k,j}=\left(\theta_{R,k,j},\phi_{R,k,j}\right)$ and $\Omega_{T,j}=\left(\theta_{T,j},\phi_{T,j}\right)$ are the AoA and angle of departure (AoD) of the *j*th path, with θ and ϕ denoting the elevation and azimuth angles, respectively. In addition, $\kappa_{k,j}$ is the channel gain of the *j*th path, modeled as

(5) $$\kappa_{k,j}=\frac{c^{2}\eta \cdot G_{T}\left(\Omega_{T,j}\right)\cdot G_{R}\left(\Omega_{R,k,j}\right)}{{\left(4\pi \right)}^{3}f_{c}^{2}d_{T,j}^{2}d_{R,k,j}^{2}}$$

where $G_{T}\left(\Omega_{T,j}\right)$ and $G_{R}\left(\Omega_{R,k,j}\right)$ are the antenna array gain of the transmitter at an angle of $\Omega_{T,j}$ and the *k*th receiver at an angle of $\Omega_{R,k,j}$, and η is the radar cross-section (RCS) of sensing objects. The frequency-domain channel $H_{k}$ can be decomposed as $H_{k}=\left[h_{k,1},h_{k,2},\ldots ,h_{k,N_{0}}\right]$, where $h_{k,n}\in C^{N_{0}\times 1}$ is the channel across the *k*th receiver and the *n*th transmit antenna in the frequency domain.

In an ISAC system, estimating the position and velocity of sensing objects is based on the estimation of the channel parameters introduced above, including $\Omega_{R,k,l}$, $d_{k,j}$ and $f_{D,k,j}$ for k=1,2,…K. It should be noted that the modulated symbol vector s should be known to all receivers so that $d_{k,j}$ and $f_{D,k,j}$ can be correctly derived. Therefore, the communication reference signal can be directly utilized for both communication and sensing purposes [21,38], while a specially designed waveform can also be used as long as the receivers reserve the waveform data [39]. All channel parameters can then be sent to a server for further joint processing, as described in Section 4.

### 2.2. Macro–Micro Cooperation

From (3) and (5), we can see that in addition to the RCS, the channel gain and consequent received signal power of the *j*th sensing object are inversely proportional to both $d_{T,j}^{2}$ and $d_{R,k,j}^{2}$. Therefore, one of the most effective ways to enhance the received signal-to-interference-plus-noise (SINR) ratio is to reduce $d_{T,j}^{2}$ and $d_{R,k,j}^{2}$, which requires the usage of microsites that can be deployed with higher density compared to macrosites. On the other hand, we wish to take advantage of macrosites that have a large transmit power and antenna aperture to provide ISAC services across a broad area. Therefore, in this work, we consider a multistatic ISAC system based on macro–micro cooperation.

**Figure 1:**
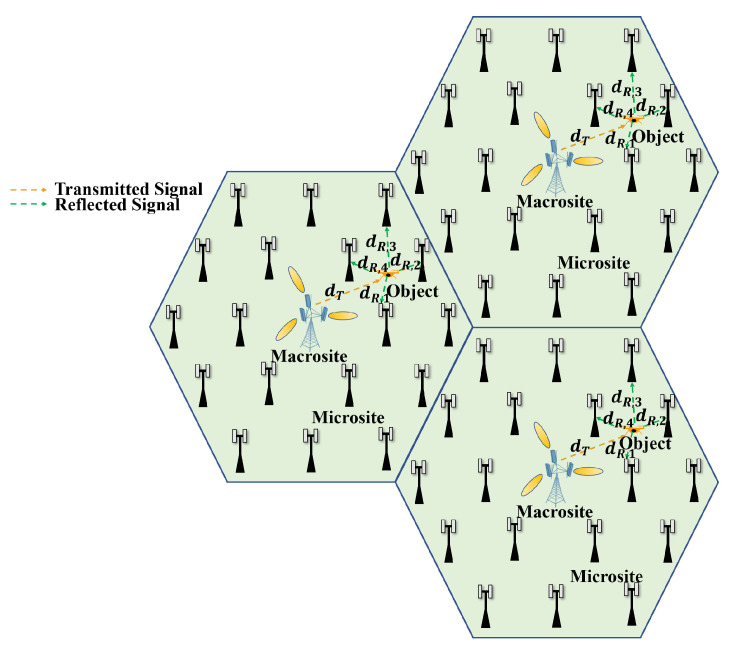
Illustration of macro–micro cooperation in a multistatic ISAC system.

As illustrated in Figure 1, microsites can be deployed around the central macrosite to fully cover the cell. When the sensing service is activated, the macrosite works as a transmitter in the downlink mode to transmit the sensing signal, while the designated microsite receivers in the uplink mode can receive the reflected signal from the sensing objects. In this way, the distance from the object to the receiver $d_{R}$ can be greatly reduced to enhance the channel gain. In contrast, in the multistatic ISAC system based on macrosite cooperation, the distance from the object to another macrosite receiver is larger than the radius of the cell, resulting in much lower received signal power and worse sensing performance. We also aim to ensure that for any sensing object location, there exist multiple surrounding microsites that can be used to detect the object’s position and velocity. Therefore, the problem of microsite deployment should be considered in the multistatic ISAC system based on macro–micro cooperation, as detailed in the next section.

## 3. Microsite Deployment

In this section, we investigate the deployment of microsites within the cell, which is critical for enhancing the performance of the multistatic ISAC system based on macro–micro cooperation.

From (5), we extract the parameters that vary in different propagation paths and define the propagation gain (PG) as

(6) $$\mathrm{PG}=\frac{G_{T}\left(\Omega_{T}\right)G_{R}\left(\Omega_{R}\right)}{d_{T}^{2}d_{R}^{2}}$$

where $d_{T}$ and $d_{R}$ determine the distance from the microsite to the macrosite, while $G_{T}$ and $G_{R}$ determine the angular distribution of microsites in the following deployment analysis. It should be noted that $G_{R}$ can be approximated as a constant since the number of receive antennas is much smaller than that of macrosites. However, since the macrosite beamsweeping range along the elevation angle is normally small, we only analyze the gain variation of the transmit antenna array on the azimuth angle, i.e., $G_{T}\left(\Omega_{T}\right)$ changes with the angle $\phi_{T}$. We aim for the PG to exceed a certain threshold ϵ, a small positive number representing the minimum desired PG. In other words, PG⩾ϵ, and it acts as the controllable factor of the propagation channel. By tuning ϵ, the resulting microsite deployment can change the minimum channel gain and the received signal power, thus achieving different sensing performance. For brevity in the following analysis, the dependence of the receiver index *k* and propagation path index *j* are not explicitly shown.

To simplify the analysis of the deployment issue, we use a circle cell with a radius of *R* instead of a hexagonal cell. We analyze a single circular cell since the microsites’ positions can be duplicated into the other sectors using symmetry. In addition, we separately analyze the deployment in the distance domain as well as the angular domain. Specifically, we divide the sector into multiple arc layers for different distances to the center and partition each arc layer into multiple strips with various central angles. To clarify the notation, we use the subscripts $\left(l,s\right)$ to denote the microsite covering the *s*th strip in the *l*th layer, where the maximum number of layers and strips are denoted by *L* and *S*.

### 3.1. Deployment in Distance Domain

**Figure 2:**
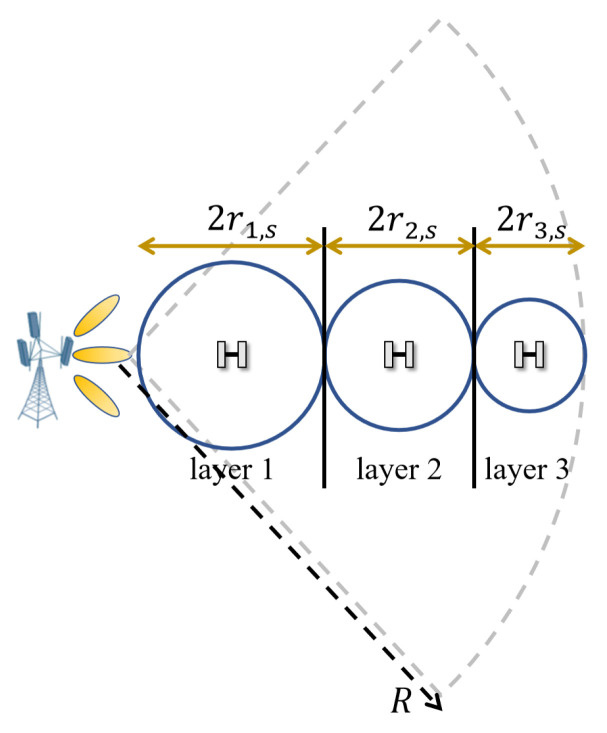
Illustration of microsite deployment (with 3 layers) along the radius of a macrosite cell.

We first analyze the distribution of microsites in the distance domain. That is, we aim to determine the number of microsites *L* placed along the radius of the cell, as shown in Figure 2. In this case, $G_{T}$ is a constant, and therefore PG is a function of $d_{T}$ and $d_{R}$.

We start by analyzing the coverage area, which has a radius of $r_{1,s}$ of the microsite in the *s*th strip of the innermost layer (referred to as layer 1 and labeled as l=1). It can be seen that the maximum distance from the macrosite to the object is

(7) $$d_{T}=\sqrt{{\left(2r_{1,s}\right)}^{2}+d_{T,y}^{2}}⩽\sqrt{{\left(2r_{1,s}\right)}^{2}+h_{T}^{2}}$$

where $2r_{1,s}$ is the maximum distance when $d_{T}$ is projected onto the azimuth plane, while $d_{T,y}$ and $h_{T}$ represent the object height and the maximum detection height with respect to the height of macrosite. Similarly, the maximum distance from the object to the microsite is given by

(8) $$d_{R}=\sqrt{{\left(r_{1,s}\right)}^{2}+d_{R,y}^{2}}⩽\sqrt{r_{1,s}^{2}+h_{R}^{2}}$$

where $r_{1,s}$ is the maximum distance when $d_{R}$ is projected onto the azimuth plane, while $d_{R,y}$ and $h_{R}$ represent the object height and the maximum detection height with respect to the height of the microsite. Therefore, we have

(9) $$\frac{G_{T}G_{R}}{\left({\left(2r_{1,s}\right)}^{2}+d_{T,y}^{2}\right)\left(r_{1,s}^{2}+d_{R,y}^{2}\right)}⩾\frac{G_{T}G_{R}}{\left({\left(2r_{1,s}\right)}^{2}+h_{T}^{2}\right)\left(r_{1,s}^{2}+h_{R}^{2}\right)}⩾\epsilon$$

so the maximum radius covered by the innermost microsite can be obtained as

(10) $$r_{1,s}=\sqrt{\frac{{\left({\left(4h_{R}^{2}-h_{T}^{2}\right)}^{2}+16\frac{G_{T}G_{R}}{\epsilon }\right)}^{\frac{1}{2}}-{\left(4h_{R}^{2}+h_{T}^{2}\right)}^{2}}{8}}.$$

With the selected $r_{1,s}$ according to (10), we proceed to analyze the microsite in layer 2, where the maximum $d_{T}$ is now given by $\sqrt{{\left(2r_{1,s}+2r_{2,s}\right)}^{2}+h_{T}^{2}}$. Similar to (9), we have

(11) $$\frac{G_{T}G_{R}}{\left({\left(2r_{1,s}+2r_{2,s}\right)}^{2}+h_{T}^{2}\right)\left(r_{2,s}^{2}+h_{R}^{2}\right)}⩾\epsilon$$

which is a quartic inequality. It is difficult to directly solve (11). However, we can see that $r_{2,s}<r_{1,s}$ due to the larger $d_{T}$. Thus, we set

(12) $$\frac{G_{T}G_{R}}{\left(4{\left(r_{1,s}+r_{2,s}\right)}^{2}+h_{T}^{2}\right)\left(r_{2,s}^{2}+h_{R}^{2}\right)}>\frac{G_{T}G_{R}}{\left(4r_{2,s}^{2}+12r_{1,s}^{2}+h_{T}^{2}\right)\left(r_{2,s}^{2}+h_{R}^{2}\right)}⩾\epsilon$$

so that the maximum range of $r_{2,s}$ can be solved as

(13) $$r_{2,s}=\sqrt{\frac{{\left({\left(4h_{R}^{2}-12r_{1,s}^{2}-h_{T}^{2}\right)}^{2}+16\frac{G_{T}G_{R}}{\epsilon }\right)}^{\frac{1}{2}}-{\left(4h_{R}^{2}+12r_{1,s}^{2}+h_{T}^{2}\right)}^{2}}{8}}.$$

By repeating the above analysis, the coverage area radius of the microsite in the *l*th layer, where l=2,…,L, satisfies

(14) $$r_{l,s}=\sqrt{\frac{{\left({\left(4h_{R}^{2}-{\left(12\sum_{\alpha =1}^{l-1}r_{\alpha ,s}\right)}^{2}-h_{T}^{2}\right)}^{2}+16\frac{G_{T}G_{R}}{\epsilon }\right)}^{\frac{1}{2}}-{\left(4h_{R}^{2}+{\left(12\sum_{\alpha =1}^{l-1}r_{\alpha ,s}\right)}^{2}+h_{T}^{2}\right)}^{2}}{8}}$$

It should be noted that the microsites’ coverage areas become smaller and the deployment density becomes higher as the number of layers increases.

The maximum number of layers *L* can then be derived when *L* satisfies

(15) $$2\sum_{l=1}^{L-1}r_{l,s}⩽R\;\mathrm{and}\;2\sum_{l=1}^{L}r_{l,s}⩾R.$$

Therefore, in the *l*th layer, the center of the microsite is placed along the radius of

(16) $$R_{l}=\left\{\begin{array}{cc} r_{1,s}, & l=1\\ 2\sum_{\alpha =1}^{l-1}r_{\alpha ,s}+r_{l,s}, & l=2,3,\ldots ,L-1\\ \frac{R}{2}+\sum_{\alpha =1}^{L-1}r_{\alpha ,s}, & l=L \end{array}\right..$$

where the center of the coverage circle of the microsite in the *L*th layer can be adjusted to the midpoint between the cell radius *R* and the point nearest to the cell edge in the $\left(L-1\right)$th layer, i.e., $2\sum_{\alpha =1}^{l-1}r_{\alpha ,s}$.

### 3.2. Deployment in Angular Domain

**Figure 3:**
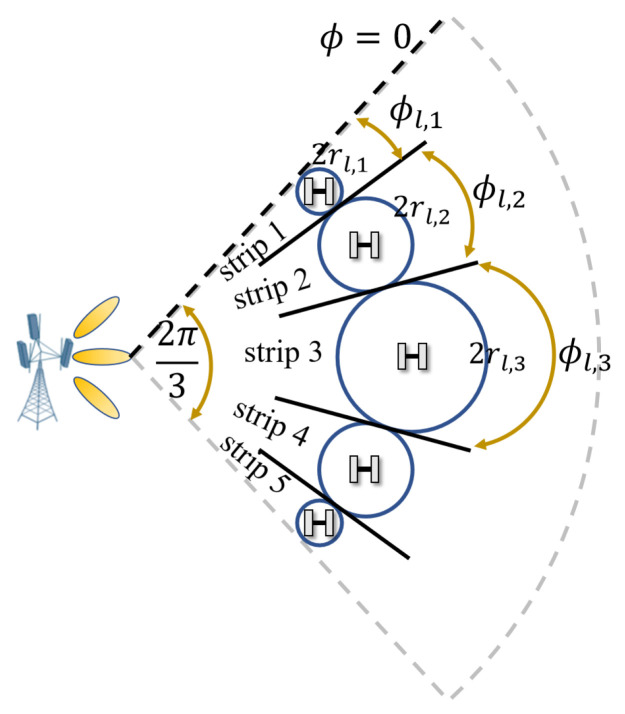
Illustration of microsite deployment (with 5 strips) over the sector angle of the macrosite cell.

Next, we analyze the distribution of microsites in the angular domain. As shown in Figure 3, we aim to derive the number of microsites $S_{l}$ placed to cover the *l*th arc layer, where $d_{T}$ can be assumed as a constant so that the coverage area radius of the microsite is a function of $G_{T}$. In addition, we analyze the microsite deployment at azimuth angles from 0 to $\frac{\pi }{3}$ (with respect to the dashed black reference line, as shown in Figure 3) since the microsite deployment at azimuth angles from $\frac{\pi }{3}$ to $\frac{2\pi }{3}$ is symmetrical about the angle $\phi =\frac{\pi }{3}$.

The coverage area radius of the microsite in the outermost strip $r_{l,1}$, referred to as strip 1 in Figure 3, can be directly obtained when ϕ=0 is assigned for $G_{T}$, i.e., $G_{T}\left(\phi =0\right)$ in (9)–(15). $r_{l,1}$ is also associated with the central angle $\phi_{l,1}$, approximated as $2\frac{r_{l,1}}{R_{l}}$. Then, we analyze the circular area with a radius of $r_{l,2}$ covered by the microsite in strip 2. It can be seen that the maximum distance from the macrosite to the object is $d_{T}=\sqrt{{\left(R_{l}+r_{l,1}\right)}^{2}+h_{T}^{2}}$. Therefore, similar to (11) and (13), we have

(17) $$\epsilon ⩽\frac{G_{T}\left(\phi =2\frac{r_{l,1}}{R_{l}}\right)G_{R}}{\left({\left(R_{l}+r_{l,1}\right)}^{2}+h_{T}^{2}\right)\left(r_{l,2}^{2}+h_{R}^{2}\right)},$$

and the maximum $r_{l,2}$ from (17) as

(18) $$r_{l,2}=\sqrt{\frac{G_{T}\left(\phi =2\frac{r_{l,1}}{R_{l}}\right)G_{R}}{\left({\left(R_{l}+r_{l,1}\right)}^{2}+h_{T}^{2}\right)\epsilon }-h_{R}^{2}}.$$

which is larger than $r_{l,1}$ since $G_{T}\left(\phi =2\frac{r_{l,1}}{R_{l}}\right)>G_{T}\left(\phi =0\right)$ for a conventional uniform antenna array with a boresight angle of $\phi =\frac{\pi }{3}$. We can also write the coverage area radius of the microsite in the *s*th strip of the *l*th layer as

(19) $$r_{l,s}=\sqrt{\frac{G_{T}\left(\phi =\frac{2\sum_{\beta =1}^{s-1}r_{l,\beta }}{R_{l}}\right)G_{R}}{\left({\left(R_{l}+r_{l,1}\right)}^{2}+h_{T}^{2}\right)\epsilon }-h_{R}^{2}}$$

The total number of strips $S_{l}$ in the *l*th layer satisfies

(20) $$S_{l}=\left\{\begin{array}{cc} 2B, & \frac{2\sum_{\beta =1}^{B-1}r_{l,\beta }+r_{l,B}}{R_{l}}<\frac{\pi }{3}\;\mathrm{and}\;\frac{2\sum_{\beta =1}^{B}r_{l,\beta }}{R_{l}}⩾\frac{\pi }{3},\\ 2B-1, & \frac{2\sum_{\beta =1}^{B-1}r_{l,B}+r_{l,B}}{R_{l}}⩾\frac{\pi }{3} \end{array}\right.$$

That is, when the azimuth angle of the coverage circle center for the microsite in the *B*th strip exceeds $\frac{\pi }{3}$, $S_{l}$ is odd by utilizing the symmetry of the sector with a central angle of $\frac{2}{3}\pi$. Otherwise, $S_{l}$ is even to ensure full coverage over the arc. In addition, utilizing the sector symmetry, we have $r_{l,\beta }=r_{l,S_{l}+1-\beta }$ for $\beta =1,2,\ldots \frac{S_{l}-1}{2}$ when $S_{l}$ is odd and for $\beta =1,2,\ldots \frac{S_{l}}{2}$ when $S_{l}$ is even.

Therefore, in the *l*th layer, $S_{l}$ microsites need to be deployed to cover the angle of the arc with a radius of $R_{l}$. The coverage circle center of the microsite along the azimuth angle can also be adjusted and written according to the parity of $S_{l}$. When $S_{l}$ is odd, the center of the $\left(l,\frac{S_{l}+1}{2}\right)$th microsite can be placed at an azimuth angle of $\phi =\frac{\pi }{3}$, and therefore $\phi_{l,s}$ is given by

(21) $$\phi_{l,s}=\left\{\begin{array}{cc} \frac{r_{l,s}}{R_{l}}, & s=1\\ \frac{2\sum_{\beta =1}^{s-1}r_{l,\beta }+r_{l,s}}{R_{l}}, & s=2,3,\ldots ,\frac{S_{l}-1}{2}\\ \frac{\pi }{3}, & s=\frac{S_{l}+1}{2}\\ \frac{2\pi }{3}-\frac{2\sum_{\beta =s+1}^{S_{l}}r_{l,\beta }+r_{l,s}}{R_{l}}, & s=\frac{S_{l}+3}{2},\frac{S_{l}+5}{2},\ldots ,S_{l}-1\\ \frac{2\pi }{3}-\frac{r_{l,s}}{R_{l}}. & s=S_{l} \end{array}\right.$$

When $S_{l}$ is even, the center of the $\left(l,\frac{S_{l}}{2}\right)$th microsite can be placed at the angle that averages $\frac{\pi }{3}$ and the overall angle covered by the first $\left(\frac{S_{l}}{2}-1\right)$ microsites in the *l*th layer, i.e., $\phi_{l,\frac{S_{l}}{2}}=\frac{1}{2}\left(\frac{\pi }{3}+\frac{2\sum_{\beta =1}^{\frac{S_{l}}{2}-1}r_{l,\beta }}{R_{l}}\right)=\frac{\pi }{6}+\frac{\sum_{\beta =1}^{\frac{S_{l}}{2}-1}r_{l,\beta }}{R_{l}}$. Thus, $\phi_{l,s}$ is given by

(22) $$\phi_{l,s}=\left\{\begin{array}{cc} \frac{r_{l,s}}{R_{l}}, & s=1\\ \frac{2\sum_{\beta =1}^{s-1}r_{l,\beta }+r_{l,s}}{R_{l}}, & s=2,3,\ldots ,\frac{S_{l}}{2}-1\\ \frac{\pi }{6}+\frac{\sum_{\beta =1}^{\frac{S_{l}}{2}-1}r_{l,\beta }}{R_{l}}, & s=\frac{S_{l}}{2}\\ \frac{\pi }{2}-\frac{\sum_{\beta =s+1}^{S_{l}}r_{l,\beta }}{R_{l}}, & s=\frac{S_{l}}{2}+1\\ \frac{2\pi }{3}-\frac{2\sum_{\beta =s+1}^{S_{l}}r_{l,\beta }+r_{l,s}}{R_{l}}, & s=\frac{S_{l}}{2}+2,\frac{S_{l}}{2}+3,\ldots ,S_{l}-1\\ \frac{2\pi }{3}-\frac{r_{l,s}}{R_{l}}. & s=S_{l} \end{array}\right.$$

### 3.3. Overall Algorithm

By combining the analyses described in Section 3.1 and Section 3.2, we have the overall algorithm to perform the microsite deployment. We first set s=1 to obtain the layer number *L* and the corresponding microsite coverage circle along the radius given ϕ=0. Then, we obtain the microsite number $S_{l}$, l=1,2,…,L along the azimuth angle in each of the *L* layers. Algorithm 1 summarizes the overall method to obtain the coverage area of microsites in the distance and angular domains. The number of layers *L* and strips $S_{l}$ in a single sector of the macrosite cell can be derived to cover the whole sector, and as a result, the exact position of microsites in polar coordinates can also obtained as $\left(R_{l},\phi_{l,s}\right)$.  
**Algorithm 1** The overall algorithm for microsite deployment.**Input:** $G_{T}\left(\phi \right)$, $G_{R}$, *R*, ϵ;   1: **Initialization:** l=1, s=1, ϕ=0;   2:    Find $r_{1,1}$ by (10);   3: **While (15) is not satisfied**        l=l+1;   4:    Find $r_{l,1}$ by (14);   5: **end While**   6: Find $R_{l}$ by (16);   7: L=l;   8: **for** l=1:L   9:     s=1;   10:   **While (20) is not satisfied**         s=s+1;   11:   Find $r_{l,s}$ by (19);   12:   **end While**   13: Find $S_{l}$ and $\phi_{l,s}$ by (20)–(22);   14: **end for****Output:** *L*, $R_{l}$, $S_{l}$, $r_{l,s}$, $\phi_{l,s}$ for l=1,2,…,L and $s=1,2,\ldots ,S_{l}$;

## 4. Multistatic Sensing

In this section, we estimate the position and velocity of sensing objects in the multistatic ISAC system with macro–micro cooperation.

For a targeted sensing area in the sector, we can select *K* microsites around the area to serve as receivers in the multistatic ISAC system. Then, by extracting the AoA, propagation distance, and Doppler velocity from the received signals of *K* microsites, we can jointly process all these channel parameters to obtain the position and velocity of objects.

### 4.1. Channel Parameter Estimation

For the received signal of the *k*th microsite $y_{k}\left(n_{c},n_{s}\right)$, we define

(23) $$g_{k}\left(\Omega ,n_{c},n_{s}\right)=a_{R,k}^{H}\left(\Omega \right)y_{k}\left(n_{c},n_{s}\right)$$

to estimate the AoA. By summing the symbol power of $g_{k}\left(\Omega ,n_{c},n_{s}\right)$ over all subcarriers and OFDM symbols for each angle Ω, we can obtain the angular power spectrum

(24) $$P_{k}\left(\Omega \right)=\sum_{n_{s}=0}^{N_{s}-1}\sum_{n_{c}=0}^{N_{c}-1}{\left|g_{k}\left(\Omega ,n_{c},n_{s}\right)\right|}^{2}.$$

The peak indices of $P_{k}\left(\Omega \right)$, denoted by ${\hat{\Omega }}_{k}=\left({\hat{\theta }}_{k},{\hat{\phi }}_{k}\right)$, with ${\hat{\theta }}_{k}$ and ${\hat{\phi }}_{k}$ being the estimated elevation and azimuth angles of objects with respect to the *k*th microsite, can be found as the AoA. This is because when $\Omega ={\hat{\Omega }}_{k}$ in (23), the steering vector in $y_{k}$ can be conjugate matched to $a_{R,k}^{H}\left({\hat{\Omega }}_{k}\right)$ so that $P_{k}\left({\hat{\Omega }}_{k}\right)$ reaches its peak.

Then, we estimate the propagation distance and Doppler velocity from $g_{k}\left({\hat{\Omega }}_{k},n_{c},n_{s}\right)$ based on the estimated AoA ${\hat{\Omega }}_{k}$. Dividing $g_{k}$ in (23) by the digital symbols on the *n*th transmit antenna, we have

(25) $$\begin{array}{cc} {\tilde{h}}_{k,n}\left({\hat{\Omega }}_{k},n_{c},n_{s}\right)= & \frac{g_{k}\left({\hat{\Omega }}_{k},n_{c},n_{s}\right)}{s_{n}\left(n_{c},n_{s}\right)}=a_{R,k}^{H}\left({\hat{\Omega }}_{k}\right)h_{k,n}\left(n_{c},n_{s}\right)+{\tilde{n}}_{k}\left(n_{c},n_{s}\right),\;n=1,2,\ldots ,N_{0} \end{array}$$

where ${\tilde{n}}_{k}\left(n_{c},n_{s}\right)=\frac{a_{R,k}^{H}\left({\hat{\Omega }}_{k}\right)\left(n_{k}\left(n_{c},n_{s}\right)+i_{k}\left(n_{c},n_{s}\right)\right)}{s_{n}\left(n_{c},n_{s}\right)}\in C^{N_{k}\times 1}$ is the ratio of interference plus noise to the transmitted symbols and ${\tilde{h}}_{k,n}\left({\hat{\Omega }}_{k},n_{c},n_{s}\right)$ is the estimated channel across the *n*th transmit antenna of the macrosite and the *k*th microsite at the AoA of ${\hat{\Omega }}_{k}$. To obtain the propagation distance and Doppler velocity, we perform (2D) discrete Fourier transform (DFT) on ${\tilde{h}}_{k,n}$ [40] as

(26) $${\tilde{H}}_{k,n}\left(p,q\right)=\frac{1}{N_{c}N_{s}}\sum_{n_{s}=0}^{N_{s}-1}\sum_{n_{c}=0}^{N_{c}-1}{\tilde{h}}_{k,n}\left({\hat{\Omega }}_{k},n_{c},n_{s}\right)e^{-i\left(p-\frac{N_{s}}{2}\right)\frac{2\pi }{N_{s}}n_{s}}e^{i\left(q-1\right)\frac{2\pi }{N_{c}}n_{c}}.$$

It should be noted that $N_{s}$ can be assumed to be an even integer without loss of generality. When ${\tilde{H}}_{k,n}$ reaches its peak, the phase term brought by the propagation distance and Doppler velocity in (3) are offset, namely

(27) $$T_{s}f_{D}=\frac{\left({\hat{p}}_{k,n}-\frac{N_{s}}{2}\right)}{N_{s}},$$

(28) $$\tau f_{\Delta }=\frac{\left({\hat{q}}_{k,n}-1\right)}{N_{c}},$$

where ${\hat{p}}_{k,n}$ and ${\hat{q}}_{k,n}$ are indices of the peak in ${\tilde{H}}_{k,n}$. Therefore, the estimated propagation distance and Doppler velocity are, respectively, given by

(29) $${\hat{v}}_{\|,k,n}=\frac{\left({\hat{p}}_{k,n}-\frac{N_{s}}{2}\right)c}{N_{s}T_{s}f_{c}},$$

(30) $${\hat{d}}_{k,n}=\frac{\left({\hat{q}}_{k,n}-1\right)c}{N_{c}f_{\Delta }}.$$

Equations (25)–(30) can be repeated for each transmitted signal stream $s_{n}\left(n_{c},n_{s}\right)$, $n=1,2,\ldots ,N_{0}$. Thus, we can average all $N_{0}$ groups of ${\hat{d}}_{k,n}$ and ${\hat{v}}_{\|,k,n}$ by ${\hat{d}}_{k}=\frac{1}{N_{0}}\sum_{n=1}^{N_{0}}{\hat{d}}_{k,n}$ and ${\hat{v}}_{\|,k}=\frac{1}{N_{0}}\sum_{n=1}^{N_{0}}{\hat{v}}_{\|,k,n}$ as the estimated propagation distance and Doppler velocity by the *k*th microsite. To this end, the estimated channel parameters $\left({\hat{\theta }}_{k},{\hat{\phi }}_{k},{\hat{d}}_{k},{\hat{v}}_{\|,k}\right)$ associated with sensing objects can be obtained. In practical implementations, these parameters can be sent to a server for data fusion to estimate the object’s position and velocity, as discussed in the next subsection.

### 4.2. Position and Velocity Estimation

In this subsection, we estimate the position and velocity of sensing objects based on (1) the channel parameters $\left({\hat{\theta }}_{k},{\hat{\phi }}_{k},{\hat{d}}_{k},{\hat{v}}_{\|,k}\right)$ for k=1,2,…,K, and (2) the positions of the macrosite and *K* microsites whose coordinates can be denoted by $\left(x_{0},y_{0},z_{0}\right)$ and $\left(x_{k},y_{k},z_{k}\right)$, respectively. Particularly, by choosing *K* microsites with polar coordinates $\left(R_{k}^{M},\phi_{k}^{M}\right)$ from (16), (21) and (22), $\left(x_{0},y_{0}\right)$ and $\left(x_{k},y_{k}\right)$ can be related by

(31) $$x_{k}=x_{0}+R_{k}^{M}\cos\phi_{k},$$

(32) $$y_{k}=y_{0}+\phi_{k}^{M}\sin\phi_{k}.$$

#### 4.2.1. Position Estimation

We first estimate the object’s position, denoted by $\left(x,y,z\right)$, by jointly processing the estimated channel parameters AoA ${\hat{\theta }}_{k}$$,{\hat{\phi }}_{k}$ and propagation distance ${\hat{d}}_{k}$, k=1,2,…,K. We aim to ensure that for each receiver, the error between the channel parameters associated with the object at location $\left(x,y,z\right)$ and the corresponding estimated channel parameters ${\hat{\theta }}_{k}$, ${\hat{\phi }}_{k}$, and ${\hat{d}}_{k}$ can be minimized. These parameters are discrete values, so we can sum the error over all *K* receivers as the loss function and formulate the optimization problem as

(33) $$\min_{x,y,z}\;\sum_{k=1}^{K}\lambda_{k}\left|{\hat{d}}_{k}-d_{T}-d_{R,k}\right|+\mu_{k}\sqrt{{\left|{\hat{\theta }}_{k}-\theta_{k}\right|}^{2}+{\left|{\hat{\phi }}_{k}-\phi_{k}\right|}^{2}}$$

where $d_{T}=\sqrt{{\left(x-x_{0}\right)}^{2}+{\left(y-y_{0}\right)}^{2}+{\left(z-z_{0}\right)}^{2}}$ is the distance between the macrosite and the object; $d_{R,k}=\sqrt{{\left(x-x_{k}\right)}^{2}+{\left(y-y_{k}\right)}^{2}+{\left(z-z_{k}\right)}^{2}}$ is the distance between the object and the *k*th microsite; and $\theta_{k}=\arccos\frac{z-z_{k}}{d_{R,k}}$ and $\phi_{k}=\arctan\frac{y-y_{k}}{x-x_{k}}$ are the elevation and azimuth angles of the object with respect to the *k*th microsite. $\lambda_{k}$ and $\mu_{k}$ are the weighting coefficients for the *k*th microsite.

For the values of $\lambda_{k}$ and $\mu_{k}$, we aim to ensure that a microsite with a small received signal power has a small weight. As per (6), the received signal power is inversely proportional to $d_{T}^{2}d_{R,k}^{2}$ [12]. Therefore, we set $\lambda_{k}=\frac{d_{T}^{-2}d_{R,k}^{-2}}{\sum_{k=1}^{K}d_{T}^{-2}d_{R,k}^{-2}}$, where $\sum_{k=1}^{K}d_{T}^{-2}d_{R,k}^{-2}$ is used to normalize the sum of $\lambda_{k}$ for k=1,2,…,K. On the other hand, the position estimation error due to the AoA estimation error can be approximated as $d_{R,k}\sqrt{{\left|{\hat{\theta }}_{k}-\theta_{k}\right|}^{2}+{\left|{\hat{\phi }}_{k}-\phi_{k}\right|}^{2}}$. Thus, we define $\mu_{k}=\frac{d_{T}^{-2}d_{R,k}^{-1}}{\sum_{k=1}^{K}d_{T}^{-2}d_{R,k}^{-1}}$, where $\sum_{k=1}^{K}d_{T}^{-2}d_{R,k}^{-1}$ is used to normalize the sum of $\mu_{k}$ for k=1,2,…,K. Then, the AoA and distance estimation errors can be added together with the same unit.

The problem in (33) is an unconstrained optimization problem. Therefore, efficient optimization algorithms, such as the quasi-Newton method [41], can be used to solve (33). To help find an optimal solution that is close to the global optimum and supports convergence, it is important to provide a good initial point for the quasi-Newton method. We can use the estimated AoA and propagation distance from a single microsite to derive the closed-form solution of the object’s position. Using the cosine theorem, the initial guess of the distance between the object and the *k*th object can be written as

(34) $$d_{R,k,\mathrm{init}}=\frac{{\hat{d}}_{k}^{2}-d_{0}^{2}}{2\left({\hat{d}}_{k}-d_{0}\cos\sigma_{k}\right)}$$

where $d_{0}=\sqrt{{\left(x_{k}-x_{0}\right)}^{2}+{\left(y_{k}-y_{0}\right)}^{2}+{\left(z_{k}-z_{0}\right)}^{2}}$ is the distance between the macrosite and the *k*th microsite and $\sigma_{k}$ is the included angle made by the line connecting the macrosite to the *k*th microsite and the line connecting the object to the *k*th microsite, which is given by

(35) $$\sigma_{k}=\frac{\langle \left[x_{k}-x_{0},y_{k}-y_{0},z_{0}-z_{k}\right],u_{k}\rangle }{d_{0}}$$

where $u_{k}$ referred to a unit vector, $u_{k}={\left[\sin{\hat{\theta }}_{k}\cos{\hat{\phi }}_{k},\sin{\hat{\theta }}_{k}\sin{\hat{\phi }}_{k},\cos{\hat{\theta }}_{k}\right]}^{T}$, of the AoA. Therefore, the initial guess of the object’s position is

(36) $$hl\left(x_{\mathrm{init}},y_{\mathrm{init}},z_{\mathrm{init}}\right)=\left(x_{k}+d_{R,k,\mathrm{init}}\sin{\hat{\theta }}_{k}\cos{\hat{\phi }}_{k},y_{k}+d_{R,k,\mathrm{init}}\sin{\hat{\theta }}_{k}\sin{\hat{\phi }}_{k},z_{k}+d_{R,k,\mathrm{init}}\cos{\hat{\theta }}_{k}\right).$$

Using the initial object position $\left(x_{\mathrm{init}},y_{\mathrm{init}},z_{\mathrm{init}}\right)$, we can derive $d_{T,\mathrm{init}}$ as the initial distance between the object and the macrosite. We can also initialize the values for $\lambda_{k,\mathrm{init}}$ and $\mu_{k,\mathrm{init}}$. With the quasi-Newton method, the optimal estimation of the object’s position can be rapidly obtained as $\left(\hat{x},\hat{y},\hat{z}\right)$.

#### 4.2.2. Velocity Estimation

Next, we estimate the object’s velocity, denoted as $v={\left[v_{x},v_{y},v_{z}\right]}^{T}$, based on the estimated object position $\left(\hat{x},\hat{y},\hat{z}\right)$ and the Doppler velocities ${\hat{v}}_{\|,k}$, k=1,2,…,K. It should be noted that 3D velocity can be recovered only when K⩾3 since each microsite receiver can only provide velocity information for a single dimension.

With the optimal estimated object position $\left(\hat{x},\hat{y},\hat{z}\right)$, we can obtain the optimal AoD as ${\hat{\theta }}_{0}=\arccos\frac{\hat{z}-z_{0}}{{\hat{d}}_{T}}$ and ${\hat{\phi }}_{0}=\arctan\frac{\hat{y}-y_{0}}{\hat{x}-x_{0}}$, with ${\hat{d}}_{T}=\sqrt{{\left(\hat{x}-x_{0}\right)}^{2}+{\left(\hat{y}-y_{0}\right)}^{2}+{\left(\hat{z}-z_{0}\right)}^{2}}$ being the distance between the estimated object position and the macrosite. The optimal AoA for the *k*th microsite is given by ${\hat{\theta }}_{k}=\arccos\frac{\hat{z}-z_{k}}{{\hat{d}}_{R,k}}$ and ${\hat{\phi }}_{k}=\arctan\frac{\hat{y}-y_{k}}{\hat{x}-x_{k}}$, with ${\hat{d}}_{R,k}=\sqrt{{\left(\hat{x}-x_{k}\right)}^{2}+{\left(\hat{y}-y_{k}\right)}^{2}+{\left(\hat{z}-z_{k}\right)}^{2}}$ being the distance between the estimated object position and the *k*th microsite. Therefore, the radial velocities $v_{\|,R,k}$ and $v_{\|,T}$ of the object with respect to the *k*th microsite and the macrosite can, respectively, be written as

(37) $$v_{\|,R,k}=u_{k}^{T}v,$$

(38) $$v_{\|,T}=u_{0}^{T}v,$$

and the Doppler velocity can be written as per (4)

(39) $$v_{\|,k}=v_{\|,R,k}+v_{\|,T}={\left(u_{k}+u_{0}\right)}^{T}v.$$

Correspondingly, the velocity optimization problem can be formulated as

(40) $$\min_{v}\;∥t^{T}\left(v_{\|}-{\hat{v}}_{\|}\right)∥,$$

where $t={\left[{\hat{t}}_{1},{\hat{t}}_{2},\ldots ,{\hat{t}}_{K}\right]}^{T}$ is the weighting coefficient vector, with ${\hat{t}}_{k}=\frac{{\hat{d}}_{T}^{-2}{\hat{d}}_{R,k}^{-2}}{\sum_{k=1}^{K}{\hat{d}}_{T}^{-2}{\hat{d}}_{R,k}^{-2}}$. $v_{\|}=U^{T}v$, where $U=\left[u_{1}+u_{0},u_{2}+u_{0},\ldots ,u_{K}+u_{0}\right]\in C^{3\times K}$ collects $u_{k}+u_{0}$, k=1,2,…,K into a matrix, and ${\hat{v}}_{\|}={\left[{\hat{v}}_{\|,1},{\hat{v}}_{\|,2},\ldots ,{\hat{v}}_{\|,K}\right]}^{T}$ collects all estimated Doppler velocities into a vector. Similar to (33), the optimization problem in (40) aims to minimize the difference between the estimated Doppler velocities ${\hat{v}}_{\|}$ and the Doppler velocities $v_{\|}$ associated with the variable v. Problem (40) is a least-square (LS) problem with the optimal analytical solution for the object’s velocity

(41) $$\hat{v}={\left[{\hat{v}}_{x},{\hat{v}}_{y},{\hat{v}}_{z}\right]}^{T}={\left(Utt^{T}U^{T}\right)}^{-1}Utt^{T}{\hat{v}}_{\|}.$$

Algorithm 2 summarizes the overall optimization method for estimating the position and velocity of sensing objects. The performance of the method is verified in the simulation section.  
**Algorithm 2** The optimization method for position and velocity estimation.**Input:** ${\hat{\theta }}_{k}$, ${\hat{\phi }}_{k}$, ${\hat{d}}_{k}$, ${\hat{v}}_{\|,k}$, k=1,2,…,K;   1: Find $\left(x_{\mathrm{init}},y_{\mathrm{init}},z_{\mathrm{init}}\right)$ using (36);   2: Find $d_{T,\mathrm{init}}$, $\lambda_{k,\mathrm{init}}$, and $\mu_{k,\mathrm{init}}$ using $x_{\mathrm{init}}$ and $y_{\mathrm{init}}$;   3: Find $\left(\hat{x},\hat{y},\hat{z}\right)$ using (33);   4: Find ${\hat{d}}_{T}$, ${\hat{d}}_{R,k}$, ${\hat{\theta }}_{0}$, ${\hat{\theta }}_{k}$, ${\hat{\phi }}_{0}$, and ${\hat{\phi }}_{k}$ using $\left(\hat{x},\hat{y},\hat{z}\right)$ in step 3;   5: Find $v_{\|,R,k}$ and $v_{\|,T}$ using (37) and (38);   6: Find $\hat{v}$ using (41);**Output:**$\left(\hat{x},\hat{y},\hat{z}\right)$ and $\hat{v}$;

## 5. Simulation Results

In this section, we simulate the performance of the proposed multistatic ISAC system by estimating the position and velocity of sensing objects. Specifically, for the simulation setup, the carrier frequency is $f_{c}=26$ GHz, where the subcarrier spacing is $f_{\Delta }=120$ kHz. The system bandwidth is BW=400 MHz, and the OFDM symbol period is $T_{s}=\frac{0.125}{14}=0.0089$ ms. Quadrature phase-shift keying (QPSK) is utilized to modulate the symbols on the subcarriers. In addition, we consider a single macrosite as a transmitter in the multistatic ISAC system, whose coverage area radius is assumed to be R=100 m. The macrosite is composed of a 16×4 antenna array, where each row has 16 antennas and each column consists of 4 antennas, which can support beamsteering in a sector with a central angle of $\frac{2}{3}\pi$. The half-wavelength separated antenna gain can be found in Chapter 5.2.3 of protocol TS 38.803 [42]. Moreover, three different antenna configurations for microsites, including 2×2, 4×2, and 8×2 antenna arrays, are considered, which are most commonly considered by operators in the construction of mobile networks since they can be fabricated with a small size and weight that flexibly fit into the environment. The heights of the macrosite and microsite in the simulation are assumed to be 25 m and 10 m, respectively. We consider a single object located in the macrosite cell with a height range of 0 to 25 m. The object’s RCS is assumed to be 1, and its velocity ranges from 5 to 35 m/s in arbitrary directions.

### 5.1. Microsite Deployment

Before evaluating the performance of the multistatic ISAC system, we first need to determine the configuration of the microsite deployment. The signal-to-interference-plus-noise ratio (SINR) is defined as

(42) $$\mathrm{SINR}=\frac{\kappa P_{T}}{N_{0}+I_{0}}$$

where $P_{T}=43$ dBm is the transmit power of the macrosite, κ is the channel gain in (5), and $N_{0}=-174+10\mathrm{lg}\mathrm{BW}$ dBm and $I_{0}=-40.2$ dBm are the noise power and interference power, respectively. Together with (5), we can determine the relationship between the threshold of the propagation gain (PG) ϵ in Section 3 and the SINR as

(43) $$\epsilon =\frac{\left(N_{0}+I_{0}\right)\mathrm{SINR}{\left(4\pi \right)}^{3}f_{c}^{2}}{P_{T}c^{2}\eta }.$$

**Figure 4:**
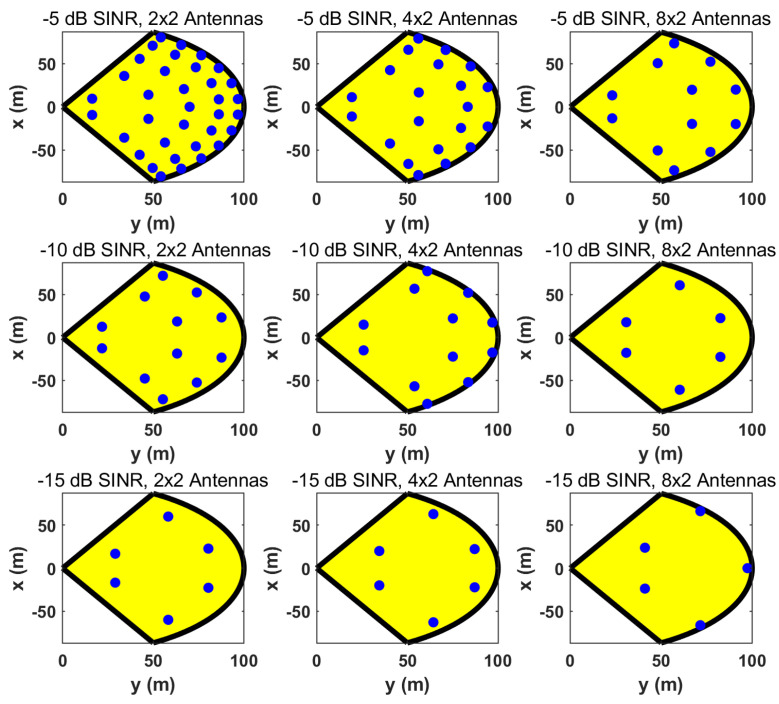
Illustration of the positions of microsites in a macrosite sector with different SINRs and numbers of antennas.

Therefore, the desired SINR determines the value of ϵ and the consequent microsite deployment. Using the proposed method in Section 3, we illustrate the positions of microsites (denoted by blue dots in a macrosite sector) with different SINRs and numbers of microsite antennas in Figure 4. It can be observed that microsite deployment becomes denser and the microsite number is higher when the desired SINR is higher or the microsite antenna number becomes smaller. However, the cost and complexity of microsite deployment are generally proportional to the number of microsites. Therefore, we aim to find the configuration that has the near-optimal sensing performance with the least number of microsites.

**Figure 5:**
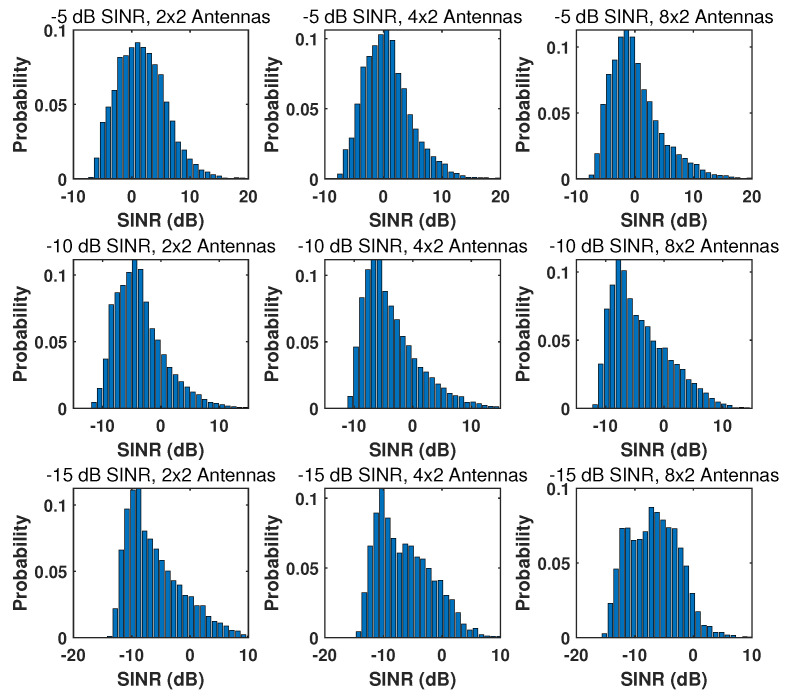
Achieved SINRs of the microsite deployments shown in Figure 4.

We also illustrate the achieved SINRs of various microsite configurations in Figure 4. For an arbitrary object position in the sector, the probability distribution of the SINRs is shown in Figure 5, where the simulated SINR values are mainly distributed above the desired SINR, verifying the microsite deployment method in Section 3.

### 5.2. Multistatic Sensing Performance

Next, we simulate the position and velocity estimation of the proposed multistatic ISAC system based on the microsite deployment described in the previous subsection. The microsites nearest to the targeted area of macrosite beamforming are selected. The system’s performance is measured by the cumulative distribution function (CDF) of the estimation error and mean estimation error. In addition, the performance of the proposed multistatic ISAC system is benchmarked against that of a multistatic ISAC system using macrosite cooperation alone, where the central macrosite in the cellular network acts as a transmitter, with its surrounding macrosites acting as the receivers, as illustrated in Figure 1. It is also benchmarked against the performance of a monostatic ISAC system, where the three macrosites in Figure 1 are assumed to support full duplexing with a 16-by-4 antenna array and ideal self-interference cancellation. The channel estimation results of the monostatic ISAC system are also jointly processed using the method described in Section 4.2. The distance between adjacent macrosites is 200 m since each macrosite covers a cell with a radius of R=100 m. Due to the sparsity of macrosites in the cellular network, we only choose three adjacent macrosites as receivers to perform multistatic or monostatic sensing.

**Figure 6:**
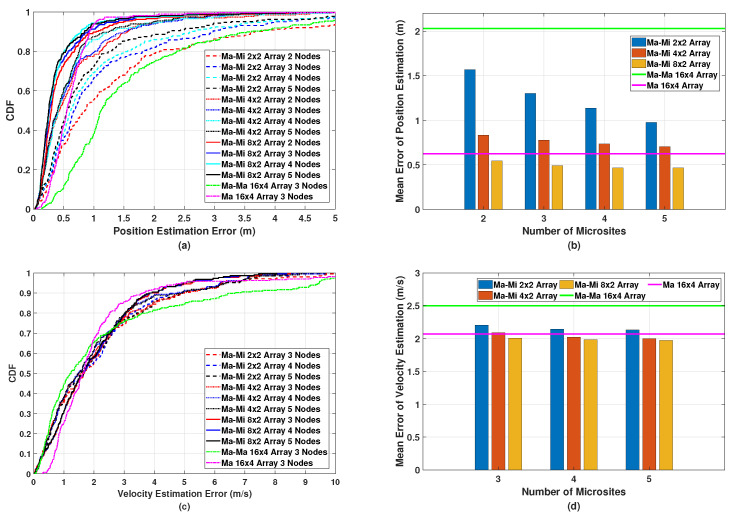
Simulated (**a**) CDF and (**b**) mean error of the position estimation, and (**c**) CDF and (**d**) mean error of the velocity estimation in the proposed multistatic ISAC system with different numbers of microsite antennas.

Based on the joint optimization method described in Section 4, we first simulate the position and velocity estimation errors in the proposed macro–micro-based multistatic ISAC system (referred to as Ma-Mi) with different numbers of microsite antennas, including 2×2, 4×2, and 8×2 antenna arrays, as shown in Figure 6. In this case, the SINR is chosen as −10 dB. For the position estimation results shown in Figure 6a,b, we can make the following observations: Firstly, by increasing the number of microsite antennas, the estimation performance can be improved since microsites with a larger number of antennas enhance the AoA estimation accuracy, which is beneficial for improving position estimation accuracy. Secondly, the estimation errors are reduced by using more microsites in multistatic sensing. This is because more microsites can estimate sensing objects from various directions and thus reduce the random estimation error of each single microsite. However, the estimation accuracy in terms of the mean error gradually converges when the number of microsites exceeds three. Thirdly, the multistatic ISAC system using macro–micro cooperation outperforms the system using macrosite cooperation alone (referred to as Ma-Ma in Figure 6), where the position estimation accuracy can be improved by over 75% using an 8×2 antenna array in the microsites. The worse performance in macrosite cooperation is due to the long distances between the objects and macrosite receivers. This also demonstrates that the proposed multistatic ISAC system using macro–micro cooperation provides better coverage for sensing service, while the efficiency of data processing is the same for both systems given the same number of receivers. Lastly, the multistatic ISAC system using macro–micro cooperation outperforms the system using monostatic sensing of full-duplex macrosites (referred to as Ma in Figure 6), further demonstrating the superiority of our proposed multistatic ISAC system. It should be noted that in practice, stringent SI cancellation is demanded in the monostatic ISAC system; otherwise, when the power difference between SI and the reflected signals from objects exceeds the maximum dynamic range of analog-to-digital converters, the reflected signals cannot be extracted from the overall received signal with SI.

In terms of velocity estimation, as shown in Figure 6c,d, the same conclusions hold: the multistatic ISAC system with microsites using an 8×2 antenna array exhibits the best performance compared to those with microsites using smaller antenna arrays, macrosite cooperation, as well as macrosite monostatic sensing. It should be noted that in practice, it is difficult to use three macrosites as receivers for cooperative sensing in a multistatic ISAC system. A key reason is that in the cellular network, all macrosites work in the same uplink or downlink mode, which cannot be easily changed.

**Figure 7:**
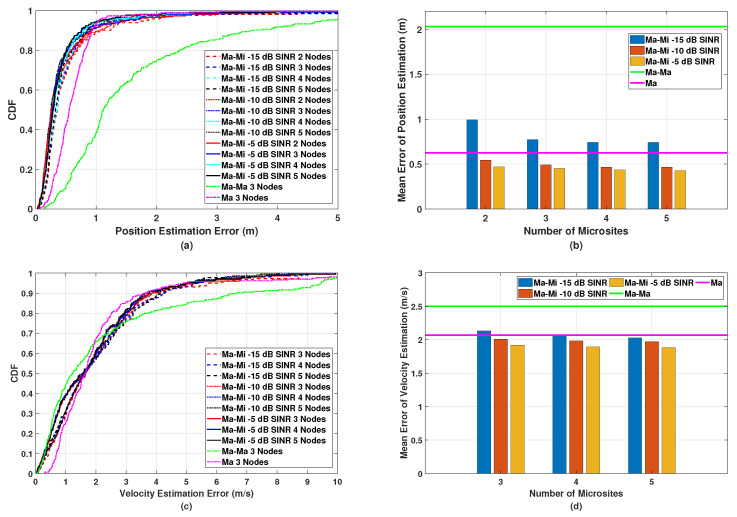
Simulated (**a**) CDF and (**b**) mean error of the position estimation, and (**c**) CDF and (**d**) mean error of the velocity estimation in the proposed multistatic ISAC system with different SINRs.

Then, we perform position and velocity estimation in the proposed multistatic ISAC system with different SINRs of −15 dB, −10 dB, and −5 dB. From Figure 6, we know that by increasing the number of microsite antennas, the estimation performance can be significantly improved. Therefore, in this simulation, the microsites are equipped with an 8×2 antenna array. The simulated results of the position estimation are shown in Figure 7a,b. It can be observed that the estimation error can be reduced by increasing the SINR value since microsites can be deployed at a higher density. However, the improvement in the estimation performance with SINR values from −10 dB to −5 dB is subtle. The CDF and mean error of the velocity estimations shown in Figure 7c,d have similar indications to those of the position estimations shown in Figure 7a,b. Therefore, it can be inferred from Figure 6 and Figure 7 that the deployment of six microsites, each with an 8×2 antenna array and an SINR value of −10 dB, achieves the highest cost performance by striking a balance between sensing performance and the number of microsites.

### 5.3. Comparison with Uniform Deployment

**Figure 8:**
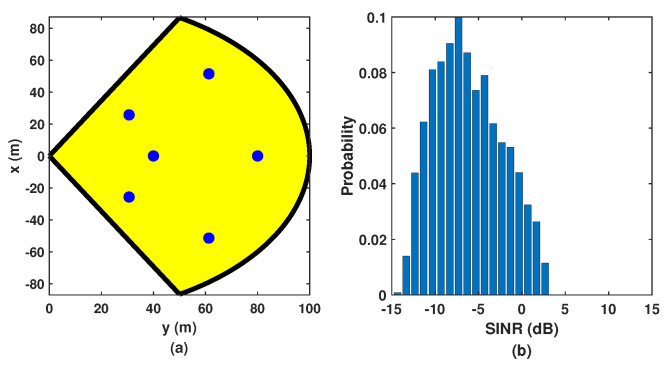
Illustration of (**a**) uniformly distributed microsites in the macrosite sector and (**b**) the achieved SINRs.

To further highlight the effectiveness of our proposed deployment method described in Section 3, we compare it with the uniform deployment strategy, in which the same number of microsites with the same 8×2 antenna array are uniformly deployed along the radius and azimuth angle in the macrosite sector, as shown in Figure 8a. Compared with the achieved SINR of the proposed deployment strategy shown in Figure 5, the simulated SINR of the multistatic ISAC system with uniformly distributed microsites shown in Figure 8b is around 3 dB smaller because the uniform deployment strategy does not consider the effect of the propagation distance and antenna array gain on the PG.

**Figure 9:**
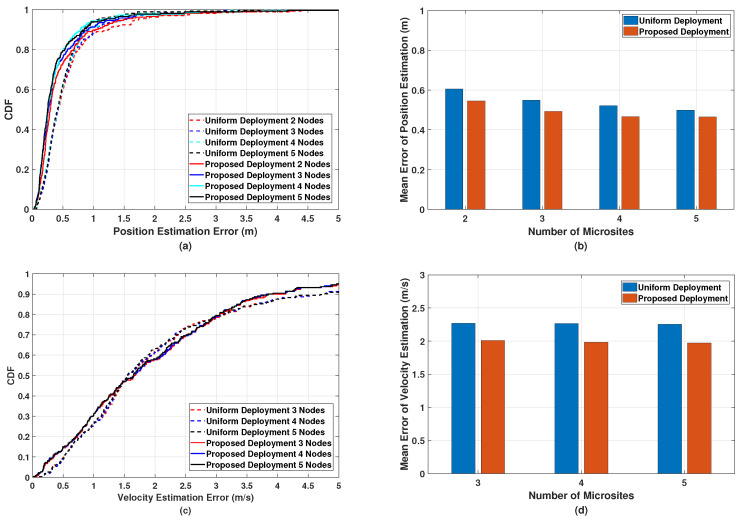
Simulated (**a**) CDF and (**b**) mean error of the position estimation, and (**c**) CDF and (**d**) mean error of the velocity estimation in the proposed multistatic ISAC system with different deployment strategies.

We also compare the position and velocity estimation performance of the multistatic ISAC system based on the uniform deployment strategy with that based on the proposed deployment strategy. In Figure 9, we can observe that given the same number of microsites, the multistatic ISAC system with the uniform deployment strategy performs worse than that with the proposed deployment strategy since in general, the relatively lower SINR leads to the degradation of estimation accuracy for channel parameters, resulting in larger position and velocity estimation errors.

To sum up, by simulating the performance of the multistatic ISAC system with different microsite configurations and deployment strategies, we can determine and verify the optimal deployment configuration that strikes a balance between sensing performance and the number of microsites. These results demonstrate the superiority of our proposed multistatic ISAC system using macro–micro cooperation compared to systems using macrosite cooperation and monostatic sensing, serving as guidance for its practical implementation in mobile networks. It should be noted that it is very challenging to use microsites at both the transmitter and receiver sides to perform multistatic sensing. This is because the transmit power of the microsite transmitter is strictly limited and is much smaller than that of macrosites, resulting in limited coverage for sensing functions. In addition, a beamsweeping procedure is normally required for object detection before formal estimation. Considering that the coverage of microsites is small, the beamsweeping range is thus too small to cover a large area, e.g., a cell sector, making microsite scheduling more complicated than cooperation between macrosites and microsites.

## 6. Discussion

### 6.1. Alternatives to Sensing Receivers

In addition to microsites, user equipment (UE) and customized sensing terminals (CSTs) can be considered as receivers in the multistatic ISAC system. We analyze the characteristics of UE and CSTs with comparisons to microsites as follows:

(1) A CST is a passive sensing receiver that can be exclusively used for sensing. Therefore, it cannot act as a transmitter for communication functions when the sensing service is not activated, leading to wasted hardware resources. On the other hand, although the hardware cost of CSTs is lower than that of microsites, both CSTs and microsites require low-latency links, such as optical fiber, for connection to the macrosites [5]. Therefore, the construction cost for CSTs is close to that for microsites. To sum up, CSTs have the potential to take the place of microsites in multistatic ISAC systems by saving hardware costs at the expense of communication functions. This tradeoff needs to be considered in practical deployments, while the deployment analysis described in Section 3 can be directly applied to multistatic ISAC systems with CSTs.
(2) UE is also an alternative to a sensing receiver. Mobile or UE-based sensing offers advantages in system extensibility, deployment cost, and implementation flexibility [43,44]. Specifically, the density of UE is much higher than that of microsites, making it more convenient to select UE closest to the targeted sensing area, while using UE as a sensing receiver almost eliminates the hardware cost for operators. However, some critical issues may arise when considering a multistatic ISAC system with UE. One issue is the synchronization between the macrosite and UE. It should be noted that a synchronization error of a few nanoseconds results in a positioning error of several meters. Therefore, difficult but stringent time and frequency offset calibrations are required. Another issue is that UE positions can drastically change, resulting in poor sensing performance. In addition, additional permission from users is needed to activate the sensing function, which may not be desired by operators. To conclude, although UE offers advantages in cost and density, some extra problems need to be addressed to improve the performance of the multistatic ISAC system.

### 6.2. Challenges

#### 6.2.1. Interference

Two major kinds of interference may arise in the proposed multistatic ISAC system. One is the sensing signal transmitted from the macrosite through the LoS path. However, the microsite receiver can use prior knowledge of the macrosite and microsite locations to calculate the propagation time. In addition, since the sensing signal is known to the receiver, the microsite can remove the signal transmitted through the LoS path directly from the received signal. Another main interference component for the microsite receiver in the proposed multistatic ISAC system is the mutual interference (MI) from adjacent microsite transmitters in the downlink communication status. However, MI is relatively low due to the smaller transmit power from the adjacent microsites, even though the interference signals may be unknown to the microsite sensing receiver. To tackle MI, beamforming or downlink power control of the microsite can be performed to reduce the interference power.

#### 6.2.2. Practical Implementation

The main challenge in the practical implementation of microsites is brought about by the possibility of complicated terrain where the calculated deployment position cannot be used for microsite construction. To address this challenge, the effect of certain terrain layouts should be taken into consideration, and substitute positions can be utilized to provide coverage, although this may require microsites with larger antenna arrays. For instance, street layouts can be considered in complex urban areas. Microsites can be mainly placed at the crossroads to facilitate coverage for vehicle-related scenarios. In this case, the optional deployment positions are discrete points in the sector instead of continuous areas, which narrows the search range in the deployment analysis.

#### 6.2.3. Power Consumption

Power consumption is also a critical challenge that needs consideration. Additional microsites become energy consumers, yet they can also provide better coverage for both sensing and communication services. Therefore, static power, i.e., power consumption when not in use, is a more important factor for overall energy saving in the mobile network. This can be realized by improving the architecture design of RF hardware, e.g., using novel analog RF architectures with low-loss tunable lumped elements replacing phase shifters that have large insertion losses [45,46]. An alternative method is based on intelligent power control. That is, a microsite could be partially shut down with few services and then woken up for normal functioning.

## 7. Conclusions and Future Work

### 7.1. Conclusions

In this paper, we propose a novel multistatic ISAC system using macrosites and microsites. The proposed system takes advantage of flexibly deployed microsites to perform multistatic sensing, where the distance between the object and the microsite receiver can be greatly reduced to enhance the received sensing signal power. Specifically, in this system, we investigate the deployment of microsites based on the characteristics of sensing path gain to cover the macrosite cell with sufficient received signal power. It can be observed that the coverage area of microsites becomes larger as the microsite locates nearer to the macrosite. By selecting multiple microsites around the targeted sensing area to extract the channel parameters related to sensing objects, joint data processing with an efficient optimization method can then be performed to obtain the object’s position and velocity. The simulation results show that the multistatic ISAC system based on macro–micro cooperation improves object position estimation accuracy by up to 75% compared to systems based on macrosite cooperation alone. It is also shown that the estimation error becomes convergent when the number of selected microsites exceeds three, which can serve as initial guidance for microsite selection in multistatic ISAC systems. The microsite configuration with high-cost performance is also provided. These results demonstrate the effectiveness of the proposed multistatic ISAC system and the potential for implementing such a system in novel application scenarios, such as UAV surveillance or autonomous driving, in the upcoming 6G mobile network.

### 7.2. Future Work

In future work, research on the multistatic ISAC system involving a non-line-of-sight (NLoS) scenario will be considered. The NLoS path components in the received signal affect the extraction of the channel parameters. Therefore, identifying the signal from the NLoS path is desired to make full use of information from the object scattering. In addition, the RCS characteristics of realistic sensing objects will be considered. The reflected signal power in different directions is determined by the RCS distribution over the surface of the object. However, the multistatic ISAC system can receive reflected signals from various angles, which is beneficial for estimating sensing objects with RCS variations. Experimental verification with data of realistic environmental conditions in novel use cases will also be performed in the future to verify the proposed system and its robustness. For different environmental conditions, the electromagnetic characteristics of the propagation channel and the interference will change, while certain terrain layouts impose additional deployment limitations on microsites. However, it should be noted that the method proposed in this work can serve as initial guidance for the deployment of microsites in mobile networks. By including real-world conditions, a more specific channel, noise, and interference model can be obtained from the measurement data in various scenarios. Then, we can incorporate these conditions into the proposed method for calculating microsite deployment. This needs to be performed in the future by us at that stage.

We will also explore the application of machine learning and AI in the multistatic ISAC system. AI is regarded as a powerful tool for 6G networks when integrated with communication, sensing, and computing. AI techniques can be helpful for rapidly performing joint data processing and generating estimation results based on the received raw signals [47]. It is also expected that estimation accuracy can be improved with AI. However, sufficient training data is a prerequisite for an accurate AI model, while obtaining such data through practical measurement using the ISAC system requires much effort and time during the training process. This also leads to the generalization and robustness issue for the AI model when encountering different sensing scenarios and random environmental changes. Proper solutions to address these challenges will enable further scalability and adaptability of AI-based multistatic ISAC systems.

## Data Availability

Restrictions apply to the availability of the data. Data were obtained from the China Mobile Research Institute and are available from the authors with the permission of the China Mobile Research Institute.
